# Soluble Fermentable Dietary Fibre (Pectin) Decreases Caloric Intake, Adiposity and Lipidaemia in High-Fat Diet-Induced Obese Rats

**DOI:** 10.1371/journal.pone.0140392

**Published:** 2015-10-08

**Authors:** Clare L. Adam, Lynn M. Thomson, Patricia A. Williams, Alexander W. Ross

**Affiliations:** Ingestive Behaviour Group, Obesity & Metabolic Health Division, Rowett Institute of Nutrition & Health, University of Aberdeen, Aberdeen AB21 9SB, Scotland, United Kingdom; National Institute of Agronomic Research, FRANCE

## Abstract

Consumption of a high fat diet promotes obesity and poor metabolic health, both of which may be improved by decreasing caloric intake. Satiety-inducing ingredients such as dietary fibre may be beneficial and this study investigates in diet-induced obese (DIO) rats the effects of high or low fat diet with or without soluble fermentable fibre (pectin). In two independently replicated experiments, young adult male DIO rats that had been reared on high fat diet (HF; 45% energy from fat) were given HF, low fat diet (LF; 10% energy from fat), HF with 10% w/w pectin (HF+P), or LF with 10% w/w pectin (LF+P) *ad libitum* for 4 weeks (*n* = 8/group/experiment). Food intake, body weight, body composition (by magnetic resonance imaging), plasma hormones, and plasma and liver lipid concentrations were measured. Caloric intake and body weight gain were greatest in HF, lower in LF and HF+P, and lowest in the LF+P group. Body fat mass increased in HF, was maintained in LF, but decreased significantly in LF+P and HF+P groups. Final plasma leptin, insulin, total cholesterol and triglycerides were lower, and plasma satiety hormone PYY concentrations were higher, in LF+P and HF+P than in LF and HF groups, respectively. Total fat and triglyceride concentrations in liver were greatest in HF, lower in LF and HF+P, and lowest in the LF+P group. Therefore, the inclusion of soluble fibre in a high fat (or low fat) diet promoted increased satiety and decreased caloric intake, weight gain, adiposity, lipidaemia, leptinaemia and insulinaemia. These data support the potential of fermentable dietary fibre for weight loss and improving metabolic health in obesity.

## Introduction

Obesity is a global public health problem and decreasing its incidence is clearly desirable. As an alternative to surgical or pharmacological treatments, the use of food ingredients to promote satiety and weight loss may offer a more physiological solution to obesity management [1, 2]. Dietary fibre is receiving increasing attention for its potential as a satiating food component [1–3] but there is a lack of experimental evidence for its long-term effects in obese subjects. Moreover, since the modern/western high fat diet is a major contributor to the widespread obesity problem, it is pertinent to investigate the dietary combination of high fibre and high fat. Rodents reared on a high fat diet develop diet-induced obesity (DIO) and provide a recognised animal model of obesity [4]. We have previously demonstrated decreased *ad libitum* food intake, adiposity and weight gain in conventional rats fed conventional low fat purified diets supplemented with soluble fermentable dietary fibre [5, 6]. The present study now examines whether one of these soluble fermentable dietary fibres (pectin) stimulates satiety and weight loss in DIO rats given high or low fat diets.

It is perhaps axiomatic that transferring DIO animals, including rats and humans, from their high fat diet onto a low fat diet would in itself lead to reduced caloric intake and decreased weight gain, but it is unknown how responses to dietary fibre supplementation of the high fat diet would compare. This is pertinent given the apparent preference for and ubiquity of high fat diets in western societies with their high incidence of obesity. There is evidence from animal models that the inclusion of soluble dietary fibre in a high fat diet protects against excessive body weight gain. Thus intake and weight gain in rats on high fat diet are decreased by supplementation with the non-viscous fermentable fibre fructoligosaccharide (FOS) [7], viscous fermentable dietary fibres (pectin, guar gum) [8,9] or highly viscous non-fermentable fibre (hydroxypropylmethylcellulose) [9], and weight gain is lowered in obesity-prone CB57 mice given high fat diet with added non-viscous fermentable inulin or viscous fermentable β-glucan [10]. Furthermore, there is evidence that dietary fibre can also improve metabolic health outcomes, by virtue of both viscosity and fermentability characteristics [11]. For example, the undesirable development of hyperlipidaemia can be counteracted in rats on high fat diet by supplementation with dietary fibre, such as fruit fibres [12], guar gum and pectin [8]. However, there is a dearth of experimental data on the effects of dietary fibre on reducing adiposity and improving lipidaemia when the animals are already obese at the start of supplementation and so the present study addresses this scenario. In addition to hyperlipidaemia, increased concentrations of the metabolic hormones insulin and leptin contribute to the metabolic ill-health of obesity and we therefore examine how they are affected by dietary fibre in DIO rats.

Apart from its effects on food intake, body weight and body composition, supplementation with the soluble fermentable dietary fibre pectin induced changes in gut morphology in our conventional rat model, notably increasing the size of small intestine and caecum [6]. In addition, plasma concentrations of the gut satiety hormones PYY and total GLP-1 were increased in parallel with the decrease in *ad libitum* food intake [6]. Here we examine in our DIO rat model how gut morphology and gut satiety hormones respond to high fat and/or high fibre diets. DIO itself is reported to influence gut morphology [13–15] and gut satiety hormones [13, 16] but it is unknown how additional dietary fibre would interact. The fibre-induced reductions in weight gain and body fat gain in our conventional rat model appeared to be attributable to decreased food intake reflecting increased satiety [5, 6] and here we investigate whether the same applies in a DIO rat model.

This study tests (and supports) the hypothesis that *ad libitum* food intake, body weight gain and adiposity are decreased in DIO rats by including soluble fermentable fibre in both high fat and low fat diets, and that some plasma and liver indices of lipidaemia and metabolic health are also improved.

## Materials and Methods

### Ethics statement

All animal experimental procedures met institutional and national guidelines for the care and use of animals. They were licensed by the UK Home Office Animals (Scientific Procedures) Act, Amended 2012, under Project License 60/4282 and were approved by the local ethical review committee at the University of Aberdeen Rowett Institute of Nutrition & Health (approval numbers SA12/17E and SA13/02E). Rats were euthanised by decapitation under general inhalation anaesthesia (isoflurane; IsoFlo, Abbott Animal Health, Maidenhead, Berkshire, UK).

### Diets

Diets were pelleted, *ad libitum*-fed, based on purified AIN-93 (American Society for Nutrition, Bethesda, MD, USA) and made and supplied by Special Diet Services Ltd, Witham, Essex, UK. The high fat diet given to all rats in the rearing period and for one group during the main experiment was a standard purified 45% energy from fat diet (HF) while the experimental low fat diet used was AIN-93M, which provides 10% energy from fat (LF). These diets contained 5–6% insoluble dietary fibre cellulose. The corresponding high fibre diets had the cellulose omitted but contained 10% soluble pectin fibre (P; high methoxyl, high viscosity apple pectin; Solgar Apple Pectin, Revital Ltd., Ruislip, Middlesex UK), thus HF with 10% w/w pectin (HF+P) and AIN-93M with 10% w/w pectin (LF+P) (Table 1).

**Table 1:** Diet composition. Composition of experimental diets (% w/w) and energy content.

	Diet			
	HF^1^	HF+P	LF^2^	LF+P
Lard^3^	17.9	19.4	0	0
Rice starch	28.3	22.8	0	0
Maize starch	0	0	46.6	41.6
Maltodextrin	0	0	15.5	15.5
Sucrose	10.5	10.5	10.0	10.0
Casein	26.5	26.7	14.0	14.0
Soyabean oil	4.3	4.3	4.0	4.0
AIN-93 Mineral mix	4.3	4.3	3.5	3.5
AIN-93 Vitamin mix	1.2	1.2	1.0	1.0
Choline bitartrate	0.30	0.30	0.25	0.25
L-cystine	0.40	0.40	0.18	0.18
Cellulose	6.2	0	5.0	0
Pectin^4^	0	10.0	0	10.0
Energy (kJ/g)^5^	18.8	18.8	15.9	15.9

Diets manufactured and supplied by Special Diet Services Ltd, Witham, Essex, UK.

^1^ AFE 45% FAT diet.

^2^ AIN-93M diet (American Society for Nutrition, Bethesda, MD USA).

^3^ Lard contains 0.97mg/g cholesterol.

^4^ Apple pectin (100% purity, high methoxyl, degree of esterification >50%; Solgar Apple Pectin; Revital Ltd., Ruislip, Middlesex UK).

^5^ Based on standard energy values of 16.7, 16.7, 37.7 and 8.4 kJ/g respectively for protein, carbohydrate, fat and fibre.

### Animals, experimental procedure and tissue collection

Sixty-four outbred male Sprague Dawley rats (Charles River Laboratories, Tranent, East Lothian, UK) were used in two independently replicated experiments. They were purchased and reared to 12 weeks of age from 6 weeks of age (Expt 1) or from 3–4 weeks of age (Expt 2) on HF diet in order to generate DIO. Then, after 1 week’s acclimatisation to individual housing in plastic cages, they were given the experimental diets *ad libitum* for 28 days (*n* = 8/diet group/experiment). Water was available *ad libitum*, the lighting regime was a standard 12 h light and 12 h dark, temperature was constant at 21±2°C and the relative humidity was held at 55±10%; cages contained sawdust bedding with shredded paper for nesting and plastic tunnels for further environmental enrichment. Voluntary food intake was measured daily by weighing uneaten food each morning and body weight was measured twice a week. Body composition was measured in conscious rats at the start (day 0) and end (28 days) of the experiment by magnetic resonance imaging (MRI; EchoMRI 2004, Echo Medical Systems, Houston, TX, USA), which provided total body fat and lean mass data.

After the final MRI scan, non-fasted rats were euthanised 1–3 h after the start of the daily light (non-eating) phase and after the end of the dark (eating) phase [17]. Final (trunk) blood samples were collected into chilled tubes containing EDTA as anti-coagulant and a peptidase inhibitor cocktail containing general protease inhibitor (cØmplete; Roche Diagnostics Ltd, Burgess Hill, West Sussex, UK) and dipeptidyl peptidase-4 inhibitor (Ile-Pro-Ile; Sigma-Aldrich, Gillingham, Dorset, UK), centrifuged immediately at 3000g for 12 min, then plasma was stored at -20°C until analysis. The gut was dissected out, wet weights were recorded immediately for stomach, small intestine, caecum and colon, and the lengths of small intestine, caecum and colon were measured. Liver samples taken from the same position and same lobe in each animal were frozen on dry ice and stored at -80°C.

### Plasma hormone analyses

Hormone concentrations in plasma samples were analysed by commercial RIA kits according to the manufacturer’s instructions (Merck Millipore, Billerica, MA, USA). Total GLP-1 was measured by kit GLP1T-36HK which detects all forms of GLP-1 (lower detection limit 3 pM). Active GLP-1 was not measured because it has a very short half-life in plasma, but measurement of total GLP-1 provides an accurate indication of overall GLP-1 secretion since it includes both the intact hormone and its primary metabolite [18]. PYY was measured by kit RMPYY-68HK (lower detection limit 15.6 pg/ml), which detects both of the circulating biologically active forms of PYY, namely PYY(1–36) and PYY(3–36). Leptin was measured by kit RL-83K (lower detection limit 0.6 ng/ml) and insulin by kit RI-13K (lower detection limit 0.08 ng/ml).

### Plasma and liver lipid analyses

Lipid concentrations in plasma were measured using a Thermo Konelab 30 Clinical Analyser with kits for total cholesterol, HDL cholesterol, triglycerides (Thermo Scientific, Thermo Fisher Scientific, Waltham, MA, USA) and non-esterified fatty acids (NEFA; Wako Chemicals GmbH, Neuss, Germany), according to the manufacturers’ instructions. Similarly, total lipids, triglycerides and total cholesterol were measured in extracted liver samples.

### Statistical methods

Daily food intakes and twice weekly body weight data were analysed by repeated measures ANOVA with time, diet, experiment and their interactions as factors (General Linear Model (GLM); Minitab Version 16, Minitab Inc., State College, PA). Group comparisons for all other data were performed by GLM ANOVA with experiment, diet and their interaction as factors. ANOVAs were followed by Tukey’s post hoc tests. Pearson’s product-moment correlation was used to examine relationships between measured parameters where indicated (Minitab). *P* values of 0.05 or less were considered statistically significant.

## Results

### Body weight and body composition

Initial body weight was greater in Expt 2 than Expt 1 (562 vs 521g, s.e.d. 10.8; *P*<0.001), reflecting Expt 2’s longer pre-experimental period on HF diet; initial total body fat (76.7 vs 63.6g, s.e.d. 5.1; *P*<0.01) and total lean mass (426 vs 389, s.e.d. 7.4; *P*<0.001) were also greater but total body fat percentage (13.5 vs 12.1%, s.e.d. 0.76) and lean tissue percentage (76.0 vs 74.7%, s.e.d. 0.74) were not significantly different. Nonetheless, mean values for these initial factors had been matched between the dietary groups across both experiments and the statistical analysis of dietary effects has been applied to the combined data.

**Fig 1:**
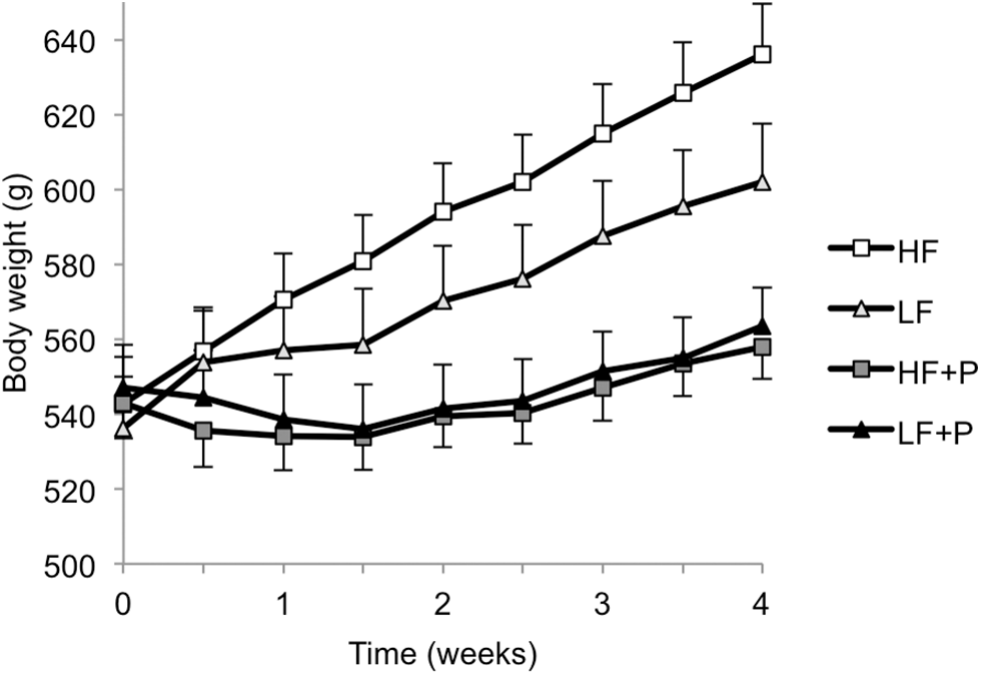
Body weight. Body weights of diet induced obese rats fed high fat diet (HF), high fat diet with 10% w/w pectin (HF+P), low fat diet (LF), or low fat diet with 10% w/w pectin (LF+P) for 4 weeks in two independently replicated experiments (*n* = 16 per diet group). ANOVA revealed HF > LF > HF+P, LF+P (*P*<0.001).

Repeated measures ANOVA revealed significant effects on body weight of diet (*P*<0.001), time (*P*<0.001) and experiment (Expt 2 > Expt 1; *P*<0.001), with no interactions, and final body weight was greatest in HF, lower in LF and lowest in both P-containing diet groups (Fig 1). The magnitude of body weight gain from start to end of dietary treatments was significantly affected by diet (in decreasing order HF > LF > HF+P and LF+P groups, *P*<0.001) but not by experiment, and there was no interaction (Table 2). Overall changes in fat mass and body fat percentage were significantly affected by diet (in decreasing order HF > LF > HF+P > LF+P groups, both *P*<0.001) and by experiment (*P*<0.01 and *P*<0.001, respectively) with significant diet x experiment interaction (*P*<0.01 and *P*<0.05, respectively) since the rats had greater fat mass at the start of Expt 2 than Expt 1 and their fat loss was greater (Table 2). Changes in total lean mass and total body lean tissue percentage were affected by diet only (*P*<0.001), with lean mass change in decreasing order HF, LF, LF+P then HF+P and lean percentage change similar in HF+P and LF+P groups, lower in LF, and lowest in HF group (Table 2). Final total body fat mass and body fat percentage were lower in all groups compared with HF (*P*<0.001), with no effect of experiment or diet x experiment interaction. Final total lean mass was affected by diet (*P*<0.001) and by experiment (Expt 2 > Expt 1; *P*<0.01), with no interaction, but final total body percentage lean was not different between diet groups and experiments, with no interaction (Table 2).

**Table 2:** Body composition and food intake. Changes in body weight and body composition, final percentage total body fat and lean, and cumulative food energy intake in diet-induced obese rats fed high fat diet (HF), high fat diet with 10% w/w pectin (HF+P), low fat diet (LF) or low fat diet with 10% w/w pectin (LF+P) for 4 weeks in two independently replicated experiments. Within rows, means with different superscript letters are significantly different (ANOVA followed by Tukey’s post hoc tests).

	Diet (*n* = 16 per group)								ANOVA		
	HF		HF+P		LF		LF+P		Significance		
	Mean	s.e.m.	Mean	s.e.m.	Mean	s.e.m.	Mean	s.e.m.	Diet	Expt	Int.
Change in:											
body weight (g)	98.0^a^	4.83	20.8^c^	4.83	70.2^b^	6.03	20.0^c^	5.74	*P*<0.001	ns	ns
total fat mass (g)	28.5^a^	1.60	-13.5^c^	3.26	3.6^b^	3.70	-26.2^d^	2.78	*P*<0.001	*P*<0.01	*P*<0.01
total body fat (%)	2.3^a^	0.27	-2.8^c^	0.50	-0.9^b^	0.48	-5.0^d^	0.39	*P*<0.001	*P*<0.001	*P*<0.05
total lean mass (g)	59.9^a^	3.22	37.0^c^	3.01	54.3^ab^	3.13	44.4^bc^	3.18	*P*<0.001	ns	ns
total body lean (%)	-2.0^c^	0.27	3.8^a^	0.43	0.2^b^	0.53	5.2^a^	0.47	*P*<0.001	ns	ns
Final:											
total body fat (%)	16.1^a^	0.79	9.5^bc^	0.61	11.7^b^	1.01	7.8^c^	0.56	*P*<0.001	*P*<0.05	ns
total body lean (%)	72.8^c^	0.72	78.5^ab^	0.56	76.6^b^	1.02	80.4^a^	0.63	*P*<0.001	*P*<0.05	ns
Food intake (MJ)	11.9^a^	0.28	9.8^c^	0.27	10.6^b^	0.33	8.4^d^	0.28	*P*<0.001	ns	ns

### Food intake

Overall cumulative food intake was equivalent in terms of mass between HF and LF diets, and lower but equivalent in HF+P and LF+P diets (625, 676, 515 and 535 g, respectively; s.e.d. 22.0, P<0.001), with no difference between experiments and no diet x experiment interaction. However, since the differences in dietary energy density would clearly impact on energy balance, food energy/caloric intake data are presented and analysed hereafter. Cumulative caloric intake was greatest in group HF, lower in LF and HF+P, and lower still in the LF+P group (*P*<0.001), with no difference between experiments and no diet x experiment interaction (Table 2). Across all groups cumulative caloric intake correlated closely with the changes in body weight (*r* = 0.77, *P*<0.001), total body fat mass (*r* = 0.81, *P*<0.001) and total body lean mass (*r* = 0.55, *P*<0.001) over the experimental period.

**Fig 2:**
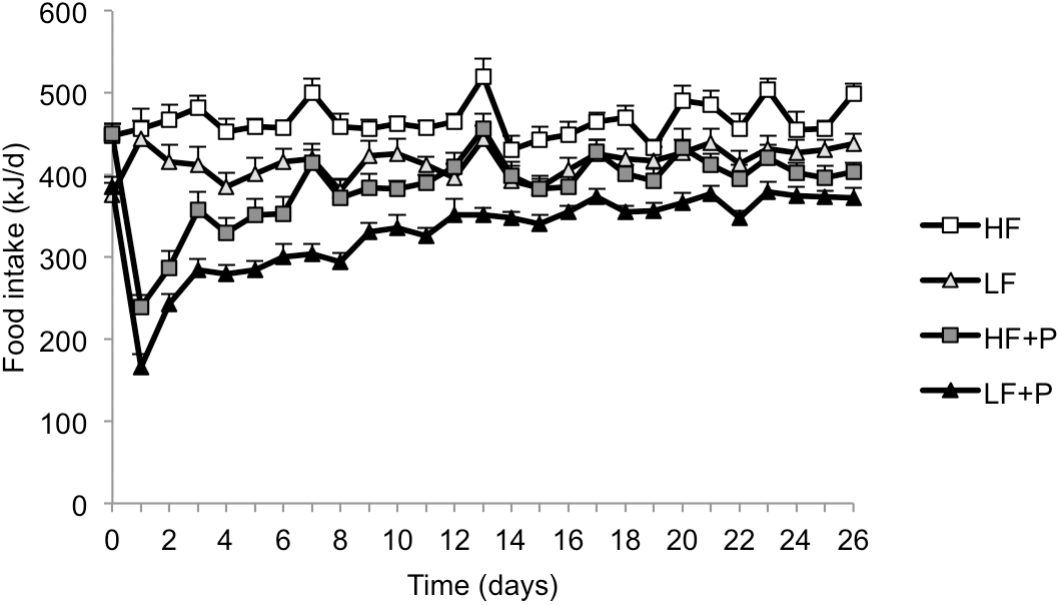
Food intake. Daily food energy intake by diet induced obese rats fed high fat diet (HF), high fat diet with 10% w/w pectin (HF+P), low fat diet (LF), or low fat diet with 10% w/w pectin (LF+P) for 4 weeks in two independently replicated experiments (*n* = 16 per diet group). ANOVA revealed HF > LF > HF+P > LF+P (*P*<0.001).

Repeated measures ANOVA revealed significant effects on daily food energy intake of diet (HF > LF > HF+P > LF+P; P<0.001) and time (*P*<0.001), but not experiment, both including and excluding the first week with exceptionally low initial intakes after the introduction of new diets (seen in both Expts); there was significant diet x time interaction (*P*<0.001) when all data were included, but this disappeared when the first week’s data were excluded (Fig 2).

### Gut morphology

There were no differences in stomach weight between the groups (Table 3). Compared with HF, small intestine and caecum weights and lengths were similar in the LF group but higher in HF+P and LF+P groups (*P*<0.001). Colon weights were not significantly different from the HF group but were higher in LF+P than LF (*P*<0.001), while there were no group differences in colon length. There were no significant differences in the results for any of these gut parameters between experiments, and no diet x experiment interactions.

**Table 3:** Gut morphology. Final gut regional weight and lengths in diet-induced obese rats fed high fat diet (HF), high fat diet with 10% w/w pectin (HF+P), low fat diet (LF) or low fat diet with 10% w/w pectin (LF+P) for 4 weeks in two independently replicated experiments. Within rows, means with different superscript letters are significantly different (by ANOVA followed by Tukey’s post hoc tests).

	Diet (*n* = 16 per group)								ANOVA		
	HF		HF+P		LF		LF+P		Significance		
	Mean	s.e.m.	Mean	s.e.m.	Mean	s.e.m.	Mean	s.e.m.	Diet	Expt	Interaction
Stomach (g)	7.2	0.70	6.1	0.34	7.4	0.34	6.2	0.47	ns	ns	ns
SI (g)	11.0^b^	0.37	13.6^a^	0.34	9.1^c^	0.26	14.4^a^	0.58	*P*<0.001	ns	ns
SI length (mm)	1101^b^	14.9	1181^a^	20.5	1071^b^	12.7	1213^a^	20.3	*P*<0.001	ns	ns
Caecum–full (g)	2.9^b^	0.25	6.9^a^	0.38	3.2^b^	0.20	8.0^a^	0.40	*P*<0.001	ns	ns
Caecum–empty (g)	1.3^b^	0.10	2.6^a^	0.14	1.2^b^	0.05	2.8^a^	0.13	*P*<0.001	ns	ns
Caecum length (mm)	37.9^b^	2.39	53.3^a^	1.79	35.7^b^	1.49	52.5^a^	1.70	*P*<0.001	ns	ns
Colon (g)	3.4^ab^	0.25	3.6^ab^	0.28	3.0^b^	0.26	4.1^a^	0.22	*P*<0.001	ns	ns
Colon length (mm)	168	2.7	167	3.8	166	2.6	171	4.8	ns	ns	ns

### Plasma hormones

**Fig 3:**
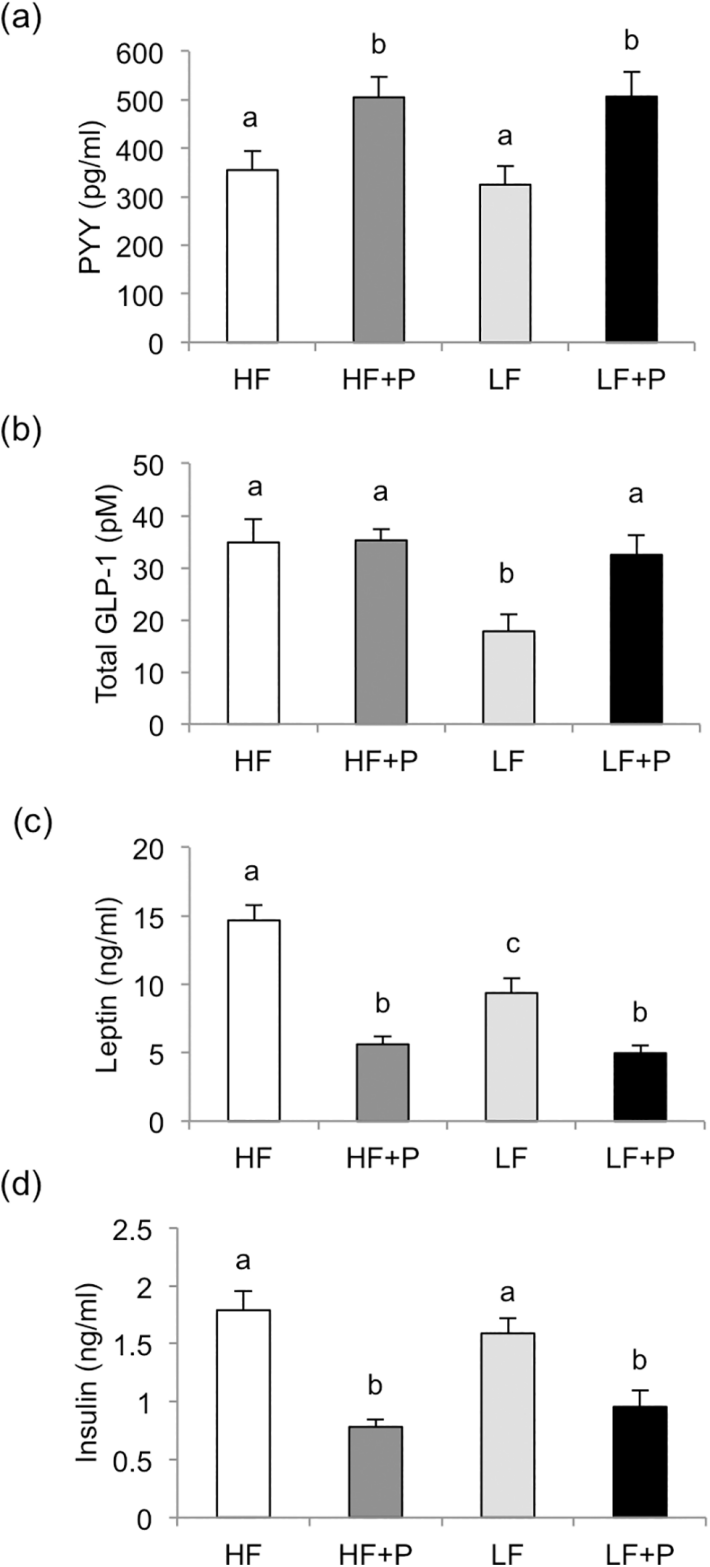
Plasma hormones. Final plasma concentrations of (a) PYY, (b) total GLP-1, (c) leptin, and (d) insulin in diet induced obese rats fed high fat diet (HF), high fat diet with 10% w/w pectin (HF+P), low fat diet (LF), or low fat diet with 10% w/w pectin (LF+P) for 4 weeks in two independently replicated experiments (*n* = 16 per diet group). Within figures, values labelled with different letters are significantly different (*P*<0.001).

Plasma PYY concentrations were affected by diet (*P*<0.001) and experiment (Expt 1 > Expt 2; *P*<0.001), with no interaction; mean values were not different between HF and LF groups but were significantly increased in both P-containing groups (Fig 3A). Plasma total GLP-1 was also affected by diet (*P*<0.001) and experiment (Expt 2 > Expt 1; *P*<0.001), with no interaction, and mean values were higher in all diet groups compared with LF (Fig 3B). Plasma leptin concentrations were influenced by diet only, with the mean values lower for the LF group than HF and lower still for both P-containing diet groups (all differences *P*<0.001; Fig 3C). Diet (*P*<0.001) and experiment (Expt 1 > Expt 2; *P*<0.01) affected plasma insulin, with no interaction; mean values were not different between LF and HF groups but were significantly decreased in both P-containing groups (*P*<0.001; Fig 3D). Across all groups, significant negative correlation was found between cumulative food energy intake and PYY (*r* = -0.32, *P*<0.01) but not between intake and total GLP-1 (*r* = -0.07). Plasma leptin and insulin were positively correlated with cumulative caloric intake (*r* = 0.72 and *r* = 0.56, respectively, both *P*<0.001) and total body fat mass (r = 0.86 and r = 0.55, respectively, both *P*<0.001).

### Plasma and liver lipids

Plasma concentrations of total cholesterol were not different between LF and HF groups but were significantly lower in both P-containing diet groups (*P*<0.001; Table 4), while HDL concentrations were not significantly different between any of the dietary groups; and there were no significant effects of experiment or diet x experiment interaction for these parameters. Plasma triglycerides were highest in the HF group, intermediate in LF, and lowest in HF+P and LF+P groups (*P*<0.001); Expt 2 values were higher than Expt 1 (*P*<0.01) and there was no diet x experiment interaction. Finally plasma NEFA concentrations were significantly lower in LF and LF+P groups than in HF and HF+P groups (*P*<0.001), with no effect of experiment and no interaction.

**Table 4:** Plasma and liver lipid status. Lipid concentrations in plasma and liver of diet-induced obese rats fed high fat diet (HF), high fat diet with 10% w/w pectin (HF+P), low fat diet (LF) or low fat diet with 10% w/w pectin (LF+P) for 4 weeks in two independently replicated experiments. Within rows, means with different superscript letters are significantly different (by Tukey’s post hoc tests).

	Diet (*n* = 16 per group)								ANOVA		
	HF		HF+P		LF		LF+P		Significance		
	Mean	s.e.m.	Mean	s.e.m.	Mean	s.e.m.	Mean	s.e.m.	Diet	Expt	Interaction
Plasma:											
Total cholesterol (mmol/l)	2.76^a^	0.136	2.15^b^	0.092	2.57^a^	0.124	2.02^b^	0.070	*P*<0.001	ns	ns
HDL cholesterol (mmol/l)	0.91	0.08	0.82	0.075	0.83	0.085	0.96	0.043	ns	ns	ns
Triglycerides (mmol/l)	2.95^a^	0.281	2.11^b^	0.273	2.41^ab^	0.297	1.36^c^	0.137	*P*<0.001	*P*<0.01	ns
NEFA (mmol/l)	0.42^a^	0.024	0.41^a^	0.029	0.28^b^	0.021	0.25^b^	0.02	*P*<0.001	ns	ns
Liver:											
Total fat (g/kg)	109.6^a^	8.87	81.4^b^	3.60	85.2^b^	7.96	54.8^c^	1.95	*P*<0.001	ns	ns
Total cholesterol (mmol/g)	11.8^a^	0.88	10.4^a^	0.64	7.48^b^	0.771	7.10^b^	0.331	*P*<0.001	*P*<0.001	ns
Triglycerides (mmol/g)	59.8^a^	8.35	39.2^b^	3.84	41.4^b^	5.77	17.8^c^	1.76	*P*<0.001	*P*<0.001	ns

Compared with the HF group, total fat and triglyceride concentrations in the liver were decreased similarly in both HF+P and LF groups, and decreased further in the LF+P group (*P*<0.001), while liver cholesterol was decreased in LF and LF+P groups (*P*<0.001; Table 4). Liver triglycerides and cholesterol were higher in Expt 2 than Expt 1, but there were no diet x experiment interactions.

## Discussion

These data support the hypothesis that caloric intake, body weight gain and adiposity are decreased in DIO rats by the addition of soluble fibre to both high fat and low fat diets, and that some plasma and liver indices of lipidaemia and metabolic health are also improved. The rats showed a normal distribution of weight gain during the pre-experimental high fat feeding period with no evidence for bimodal DIO resistance and susceptibility, in agreement with previous reports for outbred Sprague Dawley rats [19] and mimicking susceptibility to obesity in the human population.

While transfer from high to low fat diet for 4 weeks alone resulted in reduced weight gain, the addition of pectin to either diet had a much greater effect, preventing overall body weight gain altogether. These group differences were partially attributable to differences in lean tissue gain, but more striking disparities were seen in the body fat data. Thus, shifting to the low fat diet prevented the on-going further accumulation of body fat seen on the high fat diet, but the addition of dietary pectin led to loss of body fat while still on high fat diet and even greater loss when added to the low fat diet. These findings largely agree with the reported protective actions of dietary fibre against body fat gain in rats following introduction of a high fat diet [7–9] but this is the first report of dietary fibre ameliorating existing obesity in an animal model. It is of particular interest that dietary fibre is effective in this regard even as part of an “unhealthy” high fat diet.

The present data are consistent with the improvements in body weight and adiposity being attributable to the decreased caloric intake since the cumulative intake correlated closely with the changes in body weight and body fat mass across all groups. It is therefore pertinent to consider putative mechanisms of energy intake regulation. Firstly, the higher caloric intake of the high fat as opposed to low fat diet, despite no significant difference in the weight of food consumed, was attributable to the higher caloric density of the former diet; there was no evidence for underlying differences in the gut satiety hormone PYY between these diet groups. Conversely, the decreased voluntary intake (mass and energy) of the respective diets with added pectin was closely associated with increased PYY, which was indicative of increased satiety and matched earlier findings in lean rats [5, 6]. However, unlike in lean rats [5, 6], an additional satiety signalling role for GLP-1 appears equivocal in this DIO model since circulating total GLP-1 did not correlate overall with intake. Although pectin supplementation of the low fat diet did reduce intake and increase GLP-1 satiety signalling, pectin added to the high fat diet decreased intake but had no effect on GLP-1 because values were already high in the unsupplemented high fat diet group.

Central (hypothalamic) control of food intake relies on accurate feedback from the periphery not only signalling current food consumption via gut satiety hormones such as PYY but also signalling the magnitude of body energy reserves via metabolic hormones such as leptin and insulin. The present data suggest there may be a hierarchy of influence when apparently opposing signals coincide, since dietary fibre supplementation increased anorexigenic satiety hormone PYY secretion but also decreased concentrations of anorexigenic hormones leptin and insulin. Leptin and insulin are known to be inhibited in rats by imposing caloric restriction and the decreased signalling to the hypothalamus activates orexigenic neuropeptide Y (NPY) in the arcuate nucleus [20, 21] leading to the characteristic hyperphagia and rapid weight regain when the restriction is removed [22]. By contrast there was no evidence for increased orexigenic drive in the present rats since they voluntarily restricted their caloric intake despite the decreased peripheral leptin and insulin concentrations associated with their decreased adiposity. Indeed high fat-fed obesity-prone mice supplemented with the viscous fermentable fibre oat beta-glucan show suppression of arcuate NPY associated with the decreased voluntary food energy intake and increased satiety [23, 24]. Furthermore, Shen et al [25] report increased hypothalamic expression of anorexigenic pro-opiomelanocortin in rats fed dietary resistant (fermentable) starch. Clearly there is a fundamental difference between imposed and voluntary caloric restriction whereby although both scenarios decrease leptin and insulin signalling, the latter scenario does not invoke a hyperphagic response and the animals are apparently satiated. The underlying mechanism is open to speculation but may include persistence of the central leptin and insulin resistance associated with DIO [26, 27] and/or dominance of gut satiety hormone (PYY) signalling over the metabolic hormone feedback to hypothalamic appetite regulatory pathways. Nonetheless, dietary fibre-induced satiety clearly shows potential for sustained weight loss without hyperphagia and weight re-bound in the obese.

The data from this and previous studies [5, 6] are consistent with the satiety-inducing response to dietary pectin being largely attributable to its fermentability, however its high viscosity may have also contributed to the observed responses. The interaction between viscosity and fermentability of dietary fibre is clearly complex. Schroeder et al [28] reported fermentability to be important for stimulating satiety hormone PYY whereas increased viscosity was suggested to increase satiety by another mechanism such as gastric distension and/or delayed gastric emptying. In the present study, full stomach weights were not different between the groups, consistent with there being no differences in gastric distension, and data are equivocal for viscous fibres either delaying gastric emptying [29] or not [30]. Although the decreased weight gain and adiposity observed herein could largely be explained by the decreased food intake, mechanisms independent of increased satiety may also have contributed to these responses. For example, an effect of pectin viscosity *per se* cannot be ruled out since highly viscous non-fermentable fibres have been reported to limit weight gain and reduce adiposity in the absence of a significant effect on food intake in rats [28]. On the other hand, mice ingesting fermentable fibres that were either non-viscous (FOS) or viscous (guar gum) for 3 weeks exhibited decreased fat mass without showing a significant reduction in food intake [31]. Another mechanism may have included a decrease in dietary lipid digestibility, since dietary supplementation with non-viscous, fermentable FOS for 12 weeks led to decreased body fat accumulation in high fat fed mice while increasing faecal lipid excretion [32]. Moreover, the *in vitro* rate and extent of lipid digestion is apparently decreased by the addition of dietary fibres, including pectin, attributable to their physicochemical properties [33].

In addition to reducing adiposity, leptinaemia and insulinaemia, the inclusion of pectin in both low and high fat diets in the present study had a profound effect on reducing overall lipidaemia, while the effect of transfer to the low fat diet alone was arguably less dramatic at least in terms of plasma lipidaemia. Although the greatest reductions in plasma and liver lipid concentrations occurred on the low fat high fibre diet, significant improvements were also seen in rats on the high fat high fibre diet. Both pectin-containing diets decreased total cholesterol and triglycerides concentrations in plasma and total fat and triglycerides concentrations in liver, while changing from high fat to low fat diet alone led to decreased plasma NEFA concentrations (in keeping with [34]) and reduced liver total fat, triglycerides and total cholesterol. Some of these effects may have been attributable to the lower dietary intake of cholesterol in rats on low versus high fat diets and in pectin-fed rats with their overall lower food intake. Whereas earlier studies have reported the prevention of plasma and liver hyperlipidaemia following transfer to a high fat diet by supplementing with dietary fibre [8, 12, 35], the present study is the first to demonstrate an improvement in lipidaemia in rats with pre-existing high-fat diet-induced obesity. It is particularly interesting that adding pectin to the high fat diet was as effective at lowering both plasma and liver triglyceride levels as the more radical change to a low fat diet, since the former strategy may be more acceptable for dietary weight loss in obese humans. The improved dyslipidaemia associated here with the dietary fibre-induced voluntary caloric restriction and body fat loss is not seen during imposed caloric restriction and body fat loss in high fat fed rats since plasma and liver lipid concentrations remain elevated in the latter scenario [36]. Thus dietary pectin may also have influenced lipidaemia directly in the present study, in keeping with its known cholesterol-lowering properties in man which are attributed to its physicochemical characteristics, notably viscosity [37].

Dietary pectin consumed in both high and low fat diets led to increased small intestine and caecum weights in the present DIO rats, attributable to the increased dietary fermentation as seen when pectin was fed to conventional lean rats [5, 6]. There was no evidence for an effect of high fat versus low fat diet *per se* nor for the dietary fat level affecting the gut morphological response to dietary pectin. Clearly intestinal fermentation of the high fibre diets was taking place, which would have led to increased production of fermentation products, the short chain fatty acids (SCFAs). It was likely that circulating concentrations of SCFAs were also increased, which may have augmented the satiety signalling provided by circulating PYY since both acetate and propionate have been reported to act centrally in the brain to promote hypophagia [38, 39].

The ability both to consume a palatable diet to appetite and to avoid hyperphagic weight regain provides key features of a successful weight loss diet. The present data show that fermentable dietary fibre supplementation of either a high fat or a low fat diet fulfils these criteria, at least in rats, promoting decreased caloric intake and weight (fat) loss in the obese while maintaining satiation; furthermore, the increased dietary fibre intake ameliorates the obesity-associated dyslipidaemia. Although the level of dietary fibre inclusion in the present high fat diet for rats (10% w/w, or 5.3 mg/kJ) was greater than that recommended for men (3.3 mg/kJ [40, 41]), our earlier findings from lean rats indicate that lower dietary fibre (pectin) inclusion rates, up to and including 10% w/w, elicit dose-dependent satiety and weight-loss responses [6]. It is tempting to speculate that any supplementation of a high fat (or low fat) human diet with soluble fermentable dietary fibre would be beneficial for metabolic health in obesity.
